# Pragmatic Prediction in the Processing of Referring Expressions Containing Scalar Quantifiers

**DOI:** 10.3389/fpsyg.2021.662050

**Published:** 2021-08-31

**Authors:** Vinicius Macuch Silva, Michael Franke

**Affiliations:** Cognitive Modeling Group, Institute of Cognitive Science, Osnabrück University, Osnabrück, Germany

**Keywords:** pragmatics, predictive processing, pragmatic prediction, scalar quantifiers, self-paced reading, German

## Abstract

Previous research in cognitive science and psycholinguistics has shown that language users are able to predict upcoming linguistic input probabilistically, pre-activating material on the basis of cues emerging from different levels of linguistic abstraction, from phonology to semantics. Current evidence suggests that linguistic prediction also operates at the level of pragmatics, where processing is strongly constrained by context. To test a specific theory of contextually-constrained processing, termed *pragmatic surprisal theory* here, we used a self-paced reading task where participants were asked to view visual scenes and then read descriptions of those same scenes. Crucially, we manipulated whether the visual context biased readers into specific pragmatic expectations about how the description might unfold word by word. Contrary to the predictions of pragmatic surprisal theory, we found that participants took longer reading the main critical term in scenarios where they were biased by context and pragmatic constraints to expect a given word, as opposed to scenarios where there was no pragmatic expectation for any particular referent.

## 1. Introduction

Prediction in online processing has been a central theme in recent cognitive scientific and psycholinguistic research (see, inter alia, Bubic et al., 2010; Clark, 2013; Kuperberg and Jaeger, 2016). Numerous studies have shown that humans are able to predict upcoming input on the basis of perceptual, social, and linguistic cues (e.g., Kamide et al., 2003; Winkler et al., 2009; Ridderinkhof, 2017; see also Litwin and Miłkowski, 2020). As far as linguistic prediction is concerned, studies have shown that material can be pre-activated at different levels of linguistic representation, from phonologically- and lexically-driven pre-activation to pre-activation derived from syntactic and semantic cues (e.g., Mani and Huettig, 2012; Boudewyn et al., 2015; Urbach et al., 2015; Kuperberg and Jaeger, 2016), although some of these findings—particularly with respect to phonological prediction—have failed to replicate in more recent studies (e.g., Nieuwland et al., 2020; see also Kuperberg and Jaeger, 2016 for a detailed discussion of the different sorts of prediction potentially involved in predictive language processing). As for pragmatics, many studies have provided evidence that high-level semantic and pragmatic prediction occurs while people process language, from the processing of negation (e.g., Nieuwland, 2016; Haase et al., 2019; see also Scappini et al., 2015; Darley et al., 2020) to the processing of sentences containing potentially pragmatic cues such as the scalar quantifier *some* (e.g., Nieuwland et al., 2010; Augurzky et al., 2019). Although the bulk of the evidence stems from neurolinguistic studies, other studies have drawn on behavioral methods such as eye-tracking (e.g., Degen and Tanenhaus, 2016; Scholman et al., 2017; Huang and Snedeker, 2018) and self-paced reading (e.g., Bicknell and Rohde, 2009; Bergen and Grodner, 2012). Put together, the evidence suggests that people are capable of predicting linguistic as well as non-linguistic material on the basis of pragmatic and discourse-based expectations. For example, studies relying on the manipulation of the pictorial context which the linguistic stimuli refer to show that people are sensitive to contextually induced pragmatic expectation (Degen and Tanenhaus, 2016; Spychalska et al., 2016; Augurzky et al., 2019; Darley et al., 2020). In other words, there is evidence that language users form expectations about the unfolding linguistic signal based on an expectation of pragmatic felicity of the utterance in a particular visually-anchored context.

While much previous work on pragmatic processing in visually-anchored contexts has focused on EEG studies, this paper investigates whether visually-anchored pragmatic expectations also affect self-paced reading times. We use referring expressions that contain scalar quantifiers, which have been widely studied in both theoretical and experimental pragmatic research (see Huang and Snedeker, 2009; Geurts, 2010 for an overview), and which are known to act as pragmatic cues in the online processing of language. In previous research, predicatibility has been linked to the relative probability of a word serving as a continuation of an unfolding linguistic signal (Levy, 2008; Kuperberg and Jaeger, 2016). In the particular case of written language comprehension, the surprisal of a given word as a sentence continuation has been argued to be proportional to the cognitive effort of reading a word (Smith and Levy, 2013). Reading times (RTs) are thus assumed to be proportional to the effort involved in processing words in a written stream of language. Following the previous literature on visually-anchored pragmatic processing, we address *pragmatic surprisal theory*, which states that, at the level of utterance and discourse processing, language users attend to different magnitudes of pragmatic felicity and that the resulting pragmatic expectations lead to slower reading times for words with higher contextually-anchored pragmatic surprisal. To carve out concrete qualitative predictions from pragmatic surprisal theory, we assume that comprehenders are sensitive, at the very least, to the contrast between semantically congruent and semantically incongruent utterances, as well as to the contrast between pragmatically felicitous and pragmatically infelicitous utterances.

In the pragmatic tradition initiated by philosopher Paul Grice (Grice, 1975, 1989), linguistic utterances are usually analyzed, among other dimensions, in terms of their degree of informativeness, where particular terms and expressions render an utterance either more or less informative depending on other semantically-related lexical alternatives which together with the uttered term constitute a so-called linguistic scale (Horn, 1989). For our current purposes, we operationalize pragmatic felicity as informativity in the Gricean sense. Therefore, we consider a pragmatically infelicitous linguistic utterance to be a description which is underinformative, i.e., a true description for which a salient alternative exists which is also true and logically stronger, so that the latter entails the former by semantic meaning but not the other way around. While there are other aspects of pragmatic felicity, we restrict our analyses to these clearly defined benchmarks of informativity.

**Figure 1:**
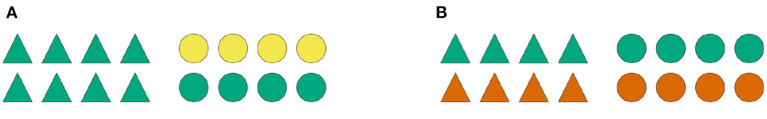
Examples of visually presented context information which raises expectations of particular lexical material based on considerations of pragmatic felicity. Scene **(A)** should raise stronger expectations than scene **(B)** when the scalar quantifier *some* is used to describe the respective visual arrays.

To illustrate how pragmatic felicity in this sense can lead to interesting predictions about expectation-based processing difficulty, consider Figure 1 (which shows example items from the experiments presented in section 2 and 3 below). Under an informativity account, an unfolding linguistic signal such as *Some of the*... may be said to be an informative description with reference to scene (a). Indeed, even though the quantified referring expression is in principle semantically congruent with either shape array depicted in the scene, from a Gricean pragmatic perspective one can expect such a description to eventually refer to the circle array and not the triangle array, given that a salient alternative expression, namely *All of the*..., would have been semantically stronger and thus pragmatically more informative in case the triangle array was the actual intended target of the referring expression. This inferential jump from a lower-bounded interpretation of *some* to an upper-bounded one is regarded as a pragmatic enrichment, more specifically a scalar implicature, whereby an interpreter is able to derive the enriched, upper-bounded meaning of the referring expression on the basis of the assumed informativity relation between the scalar alternatives *some* and *all*, which, couched in a more general expectation of pragmatic felicity, gives rise to probabilistic expectations about the to-be-mentioned shape array. Importantly, notice that a similar inference would not be possible with reference to scene (b), where neither of the shape arrays can be truthfully described using the quantifier *all*, hence there being no stronger alternatives to *some*.

Now, considering that people generate online linguistic predictions based on pragmatic expectations, their predictions should vary as a function of both the amount of linguistic information available at any given moment, i.e., how much of the unfolding linguistic signal has been processed, as well as one's overall expectations regarding what is being communicated, in this case what is being referred to. Crucially, the two are interrelated, such that expectations might shift as new material is processed and integrated into competing sentence and discourse models. In practical terms, we need to establish a link hypothesis between the predictions derived from our high-level pragmatic theory and the empirically measured processing signatures which are to reflect the effects of the postulated underlying pragmatic mechanisms. We do so in our case via surprisal, such that we expect that comprehenders will read a given description more slowly whenever their expectations fail to be met by whatever fragment of the description they are reading. That is, if a participant expects to read a specific word at a specific moment but instead reads a different word, they are expected to experience processing difficulties as a result of a mismatch between their expectations and the actual linguistic material they encounter. This is in line with previous work which has established surprisal as a possible link hypothesis in language processing, including processing as measured by means of online reading times (e.g., Demberg and Keller, 2008; Fernandez Monsalve et al., 2012; Smith and Levy, 2013).

Bringing together the theoretically-motivated considerations of pragmatic interpretation with the insights about the integration of linguistic cues in online processing via surprisal, we arrive at the following expected effects on RTs, referred henceforth as *pragmatic surprisal theory* (PST): after having read a sentence initial fragment of words *w*_1_, …*w*_*n*_, comprehenders read word *w*_*n*+1_ faster in (visual) context *C* than in context *C*′ if they expect *w*_*n*+1_ with a higher probability to occur in *C* than in *C*′. Given a quantified expression like *Some of the triangles are green*, we hypothesize that, after having read the quantifier, a comprehender will read the next critical term, i.e., the shape term, more slowly in context (b) than in context (a). This is because after processing the quantifier, more than one true and pragmatically felicitous continuation is available in (b), whereas in (a) only one true and pragmatically felicitous continuation is available (e.g., Spychalska et al., 2016; Augurzky et al., 2019). Therefore, in a scenario like (a) comprehenders are expected to generate strong predictions about a specific sentence continuation, which implies strong expectations about the to-be-read shape term in the unfolding sentence. In a scenario like (b), however, comprehenders are expected to generate weaker predictions about one or the other possible sentence continuations, which implies weaker relative expectations about the to-be-read shape term in the unfolding sentence. Whether a given prediction A or B is confirmed, participants are expected to be surprised by what they read, given that they did not have strong expectations about a given term X or Y in the first place. In short, given the cases illustrated in Figure 1, a comprehender confronted with (a) should expect to read *circles* after reading *Some of the*, as that is the most informative continuation at that point in the sentence. Crucial for our argumentation, however, this prediction should only hold when the scalar term *some* is enriched pragmatically, giving rise to a so-called scalar implicature, as discussed above.

The paper is structured as follows. Section 2 introduces the experimental material and the first part of the experiment, a sentence completion task aimed to obtain information about which descriptions participants themselves would generate for the pictorial materials. This data serves to ground our specific assumptions about which kinds of pragmatic expectations participants may have had during processing. Section 3 focuses on the second part of the experiment, a self-paced reading task, describing the design and discussing the results. Section 4 summarizes the findings and concludes.

## 2. Task 1 - Production of Referring Expressions Containing Scalar Quantifiers

In our main task, described below in section 3, we test the extent to which comprehenders process referring expressions containing scalar terms predictively on the basis of pragmatic considerations. Our working assumptions are derived from general, Gricean-inspired considerations of pragmatic felicity and informativity. More specifically, we assume that comprehenders prefer true and pragmatically felicitous utterances over true but pragmatically infelicitous utterances. However, in practice, comprehenders might have somewhat different expectations, expectations that perhaps diverge from or fuse together these different theoretically-motivated assumptions. In order to systematically tease apart pragmatic surprisal from other possible auxiliary assumptions, we first had our participants perform a production task in order to determine whether their observed production behavior gives rise to expectations that support a surprisal-based account of the reading data reported in section 3. Our aim here is to test the extent to which comprehenders' empirically verified reading patterns, assumed to be linked to their underlying pragmatic expectations, match those derived from a normative, theoretically-grounded account of pragmatic processing.

Before asking our participants to perform the reading task, we asked them to describe the same stimuli used in that task by completing sentences which had gaps in them. As explained earlier in the introduction, the aim of such task was to collect descriptions of the stimuli so as to know what the likelihood of producing specific descriptions might be in the first place. We are thus particularly interested in knowing what possible sentence configurations are more likely given the specific types of pictures found in our stimulus set.

### 2.1. Method

#### 2.1.1. Participants

Fifty-eight native speakers of German were recruited among the cognitive science and psychology student population of the University of Osnabrück. Participants were given course credit in exchange for their participation in the experiment. Data collection was conducted at the Institute of Cognitive Science of the same university, in a computer laboratory designed for the execution of behavioral experiments.

#### 2.1.2. Materials and Design

**Figure 2:**
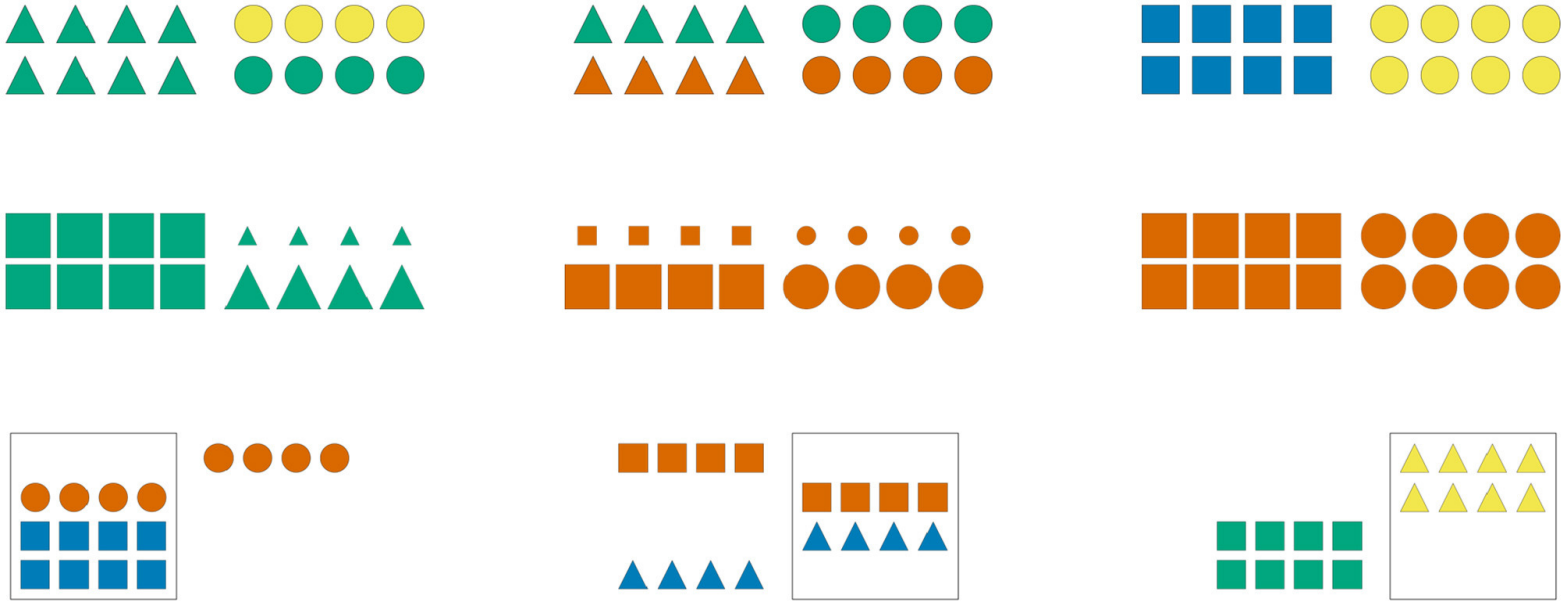
Sample visual scenes. Scenes in the first column contain one homogeneous shape array and one heterogeneous shape array; scenes in the second column contain two heterogeneous shape arrays; scenes in the third column contain two homogeneous shape arrays.

Participants were asked to complete 21 German sentences, each referring to a visual scene composed of two arrays of eight geometric shapes. Figure 2 exemplifies the different types of pictures contained in the study, which varied along three semantic dimensions: the color of the shapes (Figure 2, first row), their size (Figure 2, second row), and their position relative to a box also depicted in a subset of the scenes (Figure 2, third row).

Three choices were recorded per sentence, each of these mapping onto the critical regions from the reading task, as in the sentence structure below:


Original: QUANT | der | SHAPE |auf|dem|Bild|sind|PROP|[der|Box].

English gloss: QUANT | of the | SHAPE |in|the|picture|are|PROP|[the|box].

Each trial, participants' task was to select one scalar quantifier (QUANT), one shape term (SHAPE), and one adjective or preposition, which depending on the picture type related to a different visuo-semantic property (PROP). There were two quantifiers to choose from, three shape terms, two prepositions, and six adjectives, as shown below.

- QUANT – *einige* (some), *alle* (all);
- SHAPE – *Kreise* (circles), *Quadrate* (squares), *Dreiecke* (triangles);
- PROP – *orange* (orange), *blau* (blue), *gelb* (yellow), *grün* (green), *klein* (small), *groß* (big), *in* (inside), *neben* (next to).

#### 2.1.3. Procedure

Written as well as oral instructions were provided prior to the actual task, followed by three practice trials which mimicked the exact procedure of the test trials. Each trial, participants were presented with a visual scene as well as with sentence with gaps in it. Participants were instructed to fill the gaps in the sentence by choosing between different words in drop-down menus available on screen. After making their choices and filling all gaps, a “next” button appeared on screen, allowing participants to proceed to the next trial.

### 2.2. Results and Discussion

**Figure 3:**
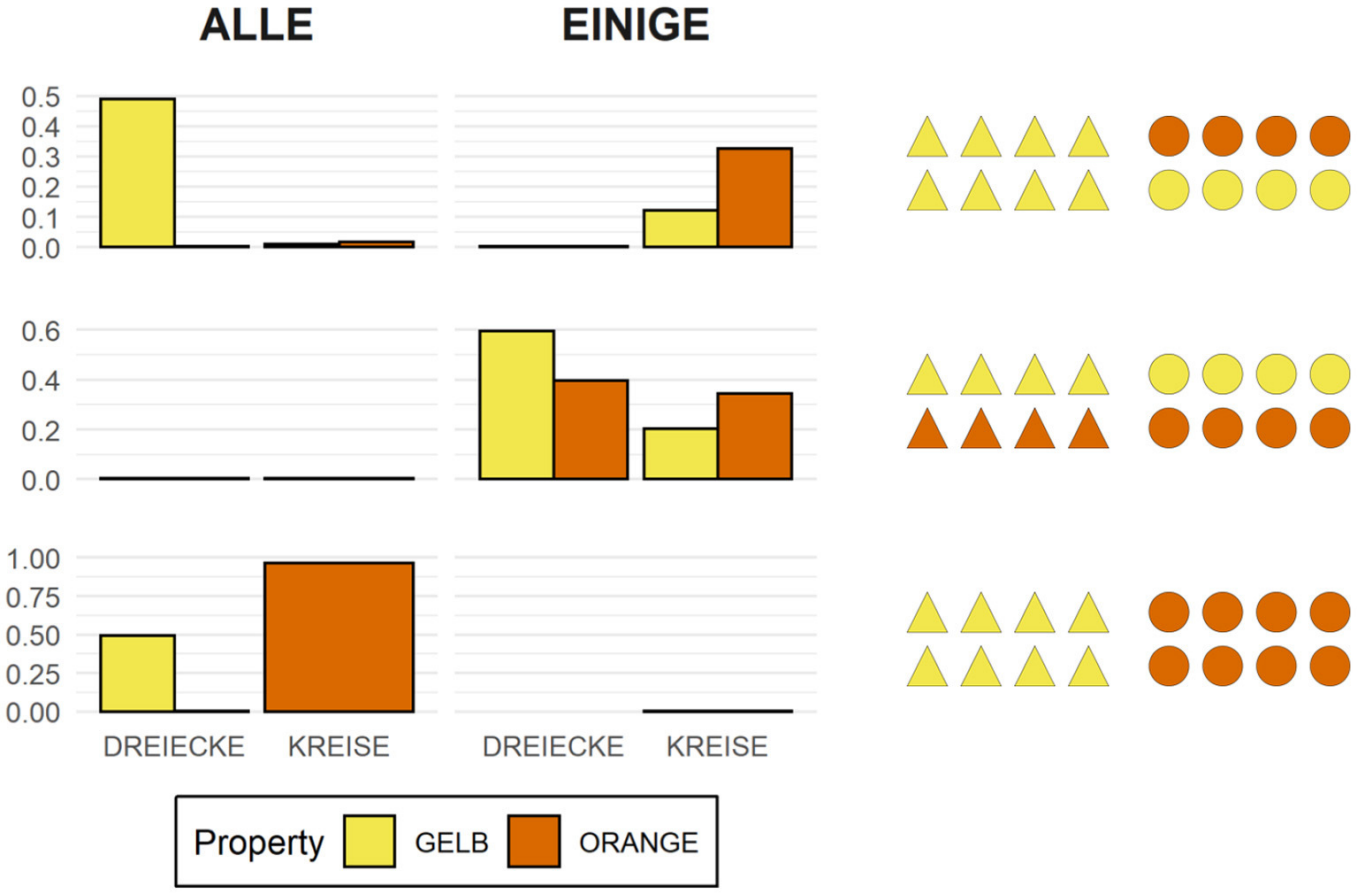
Proportion of production choices by picture type. The written labels indicate the type of expression selected by participants, which included a quantifier (*alle* vs. *einige*), a shape term (*Dreiecke* vs. *Kreise*), and a property term (*gelb* vs. *orange*). The left column shows descriptions containing the quantifier *alle*, while the right column shows descriptions containing the quantifier *einige*.

Recall that for every test sentence participants chose a quantifier, a shape term, as well as a third term which, depending on the type of picture, could either be an adjective denoting color or size or a preposition. Figure 3 shows the proportion of produced combinations of quantifier + shape term + property term, anchored to sample items where color is the relevant property to be described. Each row shows the production preferences for a given type of visual scene, the left column showing the choices of expressions containing the quantifier *alle*, and the right column showing the choices of expressions containing the quantifier *einige*. The color of the bars represent the respective color term used in the descriptions.

Unsurprisingly, the results show that participants produced descriptions of pictures with two homogeneous arrays [bottom row] virtually always using the quantifier *alle* (left column), much the same way that they produced descriptions of pictures with two heterogeneous arrays [middle row] virtually always using the quantifier *einige* (right column). This is in line with the naive assumption that people prefer semantically congruent utterances over semantically incongruent ones. The results also show that, when describing pictures with only one heterogeneous array [top row], participants prefer producing descriptions containing the quantifier *alle*, that is, they prefer referring to the homogeneous array in the scene. This can be interpreted as a bias for producing descriptions using terms which are semantically stronger, and thus pragmatically more informative, than their scalar alternatives.

Another bias participants show when describing pictures with only one heterogeneous array is to prefer referring to the semantic dimension which is congruent exclusively with the heterogeneous array. In other words, when people use *einige* to refer to a scene like the one depicted on the top row of Figure 3, they tend to couple the quantifier with a property term that renders the description maximally distinct from other possible descriptions of that same picture, such as *Einige der Kreise auf dem Bild sind* *orange* (English: Some of the circles in the picture are orange). Both this preference as well as the preference for semantically strong lexical alternatives are in line with the naive assumption that, all things being equal, people prefer pragmatically felicitous utterances over pragmatically infelicitous ones. Yet, despite these preferences, the question remains as to whether the normative expectations supported by such production data are borne out in the processing data.

## 3. Task 2 - Processing of Referring Expressions Containing Scalar Quantifiers

### 3.1. Method

#### 3.1.1. Materials and Design

Participants read 84 German sentences, each referring to a varying visual scene, as per the stimuli in Task 1. Participants saw four instances of each picture type for each condition, for a total of 84 trials. Reading times were measured at eight to ten sentence regions, each of these consisting of one word of the same sentence structure introduced above:


QUANT | der | SHAPE |auf|dem|Bild|sind|PROP|[der|Box].

In the reading task, the terms represented by uppercase words varied each trial. Much like in Task 1, each sentence contained a scalar quantifier (QUANT), a shape term (SHAPE), and either an adjective denoting color or size or a preposition (PROP):

- QUANT – *einige* (some), *alle* (all);
- SHAPE – *Kreise* (circles), *Quadrate* (squares), *Dreiecke* (triangles);
- PROP – *orange* (orange), *blau* (blue), *gelb* (yellow), *grün* (green), *klein* (small), *groß* (big), *in* (inside), *neben* (next to).

Different sentences and pictures were paired so as to yield four critical experimental conditions. These conditions differ in terms of the quantifier which was read as well as in terms of whether the matching visual scene induced specific linguistic expectations at the SHAPE region. In other words, the experimental manipulation involved modulating the predictability of the shape terms by means of varying the visual context which participants encountered immediately before reading a test sentence. We take predictability in our task to be primarily dependent on pragmatic considerations of a Gricean nature, as described above, such that readers are expected to find a given critical term more or less predictable given the assumption that a referring expression produced by a cooperative describer is informative. As such, the resulting experimental conditions are as follows:

- Alle (Biased) - Sentences containing the quantifier *alle* were paired with visual scenes which were meant to increase the predictability of specific critical terms (i.e., one of the arrays is homogeneous while the other is heterogeneous—the contrast should bias the expectation of the critical terms which match the homogeneous array). A sample pairing is as follows:

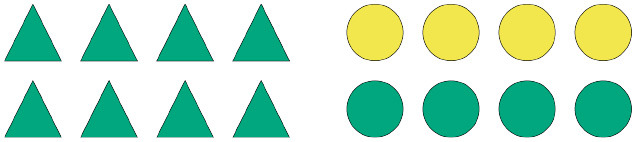

  Original: “Alle der Dreiecke auf dem Bild sind grün.”
  English gloss: “All of the triangles in the picture are green.”
  Upon reading *alle*, an interpreter who is expecting a semantically congruent utterance should predict the description to refer to the triangle array, and not the circle array, as only in the case of the former is the description congruent with the scene.
- Alle (Unbiased) - Sentences containing the quantifier *alle* were paired with visual scenes which were meant not to increase the predictability of any subsequent critical term (i.e., both arrays are homogeneous). A sample pairing is as follows:

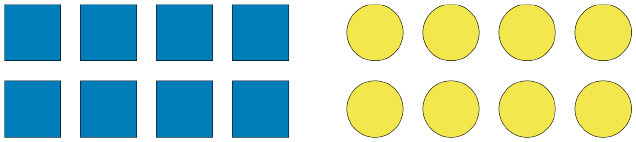

  Original: “Alle der Kreise auf dem Bild sind gelb.”
  English gloss: “All of the circles in the picture are yellow.”
- Einige (Biased) - Sentences containing the quantifier *einige* were paired with visual scenes which were meant to increase the predictability of specific critical terms (i.e., one of the arrays is heterogeneous while the other is homogeneous—the contrast should bias the expectation of the critical terms which match the heterogenous array, however, only if *einige* is read as *some-but-not-all*). A sample pairing is as follows:

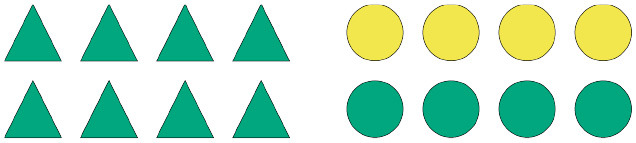

  Original: “Einige der Kreise auf dem Bild sind gelb.”
  English gloss: “Some of the circles in the picture are yellow.”
  While in the case of the Alle (Biased) condition the sentence bias originates from an expectation of truthfulness, in the case of Einige (Biased) the bias originates from an expectation of informativeness, which, according to the inferential account we are entertaining, should give rise to an implicature.
- Einige (Unbiased) - Sentences containing the quantifier *einige* were paired with visual scenes which were meant not to increase the predictability of any subsequent critical term (i.e., both arrays are heterogeneous). A sample pairing is as follows:

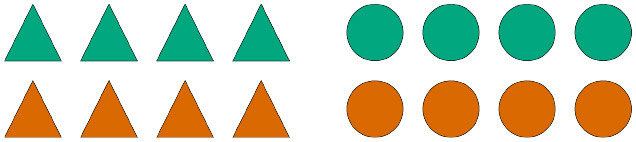

  Original: “Einige der Kreise auf dem Bild sind orange.”
  English gloss: “Some of the circles in the picture are orange.”

In addition to the four critical conditions, the experiment includes three conditions which serve as a baseline for the manipulations involving pragmatic expectations. These consist of pairings of sentences and pictures which resulted in semantically incongruent descriptions, as well as a condition in which descriptions containing the quantifier *einige* are semantically congruent but underspecified:

- Alle (False) - Sentences containing the quantifier *alle* were paired with visual scenes which are semantically incongruent with the referring expression (i.e., the referred array is not homogeneous). A sample pairing is as follows:

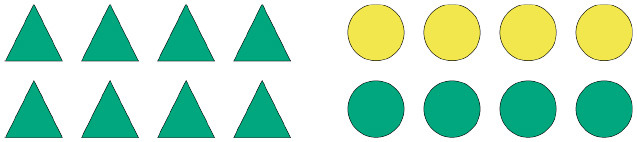

  Original: “Alle der Kreise auf dem Bild sind gelb.”
  English gloss: “All of the circles in the picture are yellow.”
- Einige (False) - Sentences containing the quantifier *einige* were paired with visual scenes which are semantically incongruent with the referring expression (i.e., both arrays are heterogeneous but the shape term renders the description semantically incongruent). A sample pairing is as follows^1^:

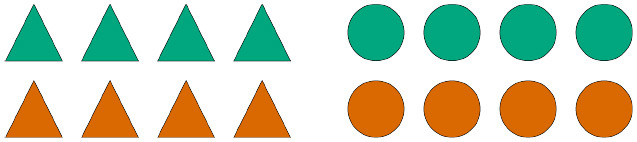

  “Einige der Quadrate auf dem Bild sind orange.”
- Einige (Infelicitous) - Sentences containing the quantifier *einige* were paired with visual scenes which were meant to increase the predictability of specific critical terms (i.e., one of the arrays is heterogeneous while the other is homogeneous—the contrast should bias the expectation of the critical terms which match the heterogenous array). A sample pairing is as follows:

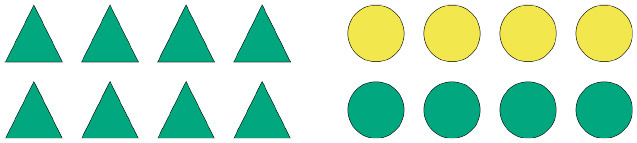

  “Einige der Dreiecke auf dem Bild sind grün.”
  English gloss: “Some of the triangles in the picture are green.”
  Note that in this condition the description, which contains the quantifier *einige*, refers to the homogeneous array, which implies that if the utterance is ultimately interpreted as a congruent description of the scene no pragmatic enrichment must have taken place, suggesting a strictly semantic interpretation of the quantifier *einige*, meaning *some-and-maybe-all*.

In summary, the study contains seven experimental conditions—four critical conditions and three control conditions—as schematized in Table 1.

**Table 1:** Study design.

	**Quantifier**	
	***Alle***	***Einige***
Bias	Alle (Biased)	Einige (Biased)
	Alle (Unbiased)	Einige (Unbiased)
Control	Alle (False)	Einige (False)
		Einige (Infelict)

#### 3.1.2. Procedure

Written as well as oral instructions were provided to participants prior to the task, followed by three practice trials which mimicked the exact procedure of the test trials ^2^. All trial elements flashed in and out of the screen in a pre-specified order. First, a fixation cross appeared at the center of the screen. After 500 ms, the cross disappeared and the visual scene became visible at the cross location. The picture remained on screen for as long as participants wished, and it was dismissed by a press of the space bar. Immediately after the picture disappeared, underscores appeared below the picture location, along with the written indication “Press the SPACE bar to reveal the words,” presented in German. As indicated by the cue, each press of the space bar revealed one of the sentence chunks foreshadowed by the underscores. Once participants reached the last chunk in the sentence, their next key press triggered the question “How accurate was the sentence as a description of the picture?”, presented in German. As indicated by the question, participants' task was to rate, on a 7-point scale, how appropriate the sentence was as a description of the visual display they saw on screen. After making their choice, participants were forwarded to the next trial.

There were 14 trials of each trial type and all participants saw all of these, for a total of 84 trials. Given the constraints imposed by the experimental manipulations, similar or even identical visual scenes were paired with different referring expressions. However, the matching between an image and a description always resulted in unique trial instantiations. The 84 trials were administered in four blocks of 21 trials. In between blocks, participants encountered a pause screen, and they were encouraged to take as much time as needed before proceeding to the next block.

### 3.2. Hypotheses

**Figure 4:**
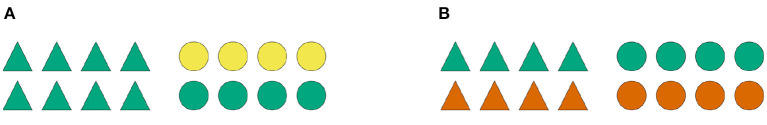
Biased **(A)** and unbiased **(B)** scenarios for descriptions containing the quantifier *einige*.

Given the design of the study and the considerations outlined in the introduction, we put forward the following general prediction: participants will read the critical terms more slowly if they are unexpected. Thus, we hypothesize that at the shape region, i.e., the next critical region after the quantifier, participants will read the critical term more slowly in the unbiased conditions than in the biased conditions, regardless of the quantifier they encounter. This is under the assumption that, after processing the quantifier, more than one informative continuation is available per scene in the unbiased scenarios, whereas in the biased scenarios only one informative continuation is available. Therefore, participants in the biased conditions are expected to generate strong predictions about a specific sentence continuation, which implies strong expectations about the to-be-read shape term in the unfolding sentence. In the unbiased conditions, however, participants are expected to generate weaker predictions about one or the other possible sentence continuations, which implies weaker relative expectations about the to-be-read shape term in the unfolding sentence. Whether a given prediction A or B is confirmed, participants are expected to be surprised by what they read, given that they did not have strong expectations about a given term X or Y in the first place. Figure 4 shows what a biased and an unbiased scenario look like in relation to a description containing the quantifier *einige*.

Considering the cases illustrated in Figure 4, a participant confronted with (a) should expect *Kreise* (English: circles) after reading *Einige der* (English: Some of the), as that is the most informative continuation at that point in the sentence. This prediction should only hold, however, when the quantifier *einige* is taken as a pragmatic, as opposed to a semantic, cue. Thus, we expect that the bias manipulation will yield different results depending on whether participants process the sentences as pragmatically enriched or strictly semantic descriptions. Namely, we expect that when *einige* is interpreted as *some-and-maybe-all* reading times will be similar in both the biased and unbiased conditions, as in both cases participants are expected not to generate strong predictions about one or the other possible sentence continuations.


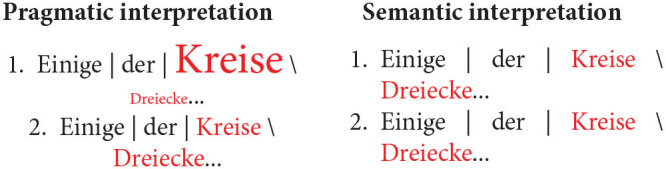


We assume that, after processing the quantifier and the shape term, participants will then generate predictions about the last semantically relevant term in the sentence. Considering again the cases in Figure 4, we may assume that in the unbiased scenario, even if participants already know the shape term, it is still unclear which color might be referred to. Much the same way, in the biased scenario, even if participants already know that the sentence refers to circles, the color of the referred array cannot be easily predicted. In fact, even if *einige* is taken as a pragmatic cue (*some-but-not-all*), participants should not be able to generate strong predictions about a particular color term.


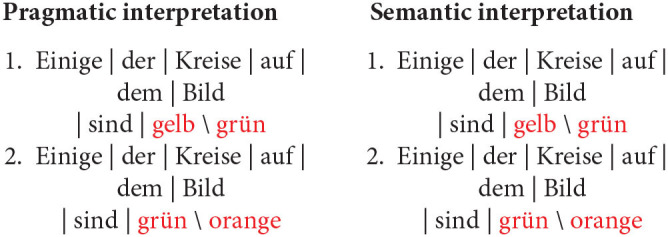


In summary, then, the following predictions are generated from our surprisal-based account of pragmatic prediction:

1. At the shape term, descriptions will be read more slowly in the unbiased conditions compared to the biased conditions. This will be the case for descriptions containing *einige* only if comprehenders interpret the quantifier as a pragmatic cue;
2. At the shape term, descriptions containing *einige* will be read equally fast in both conditions, if comprehenders interpret *einige* as a semantic cue.

**Figure 5:**
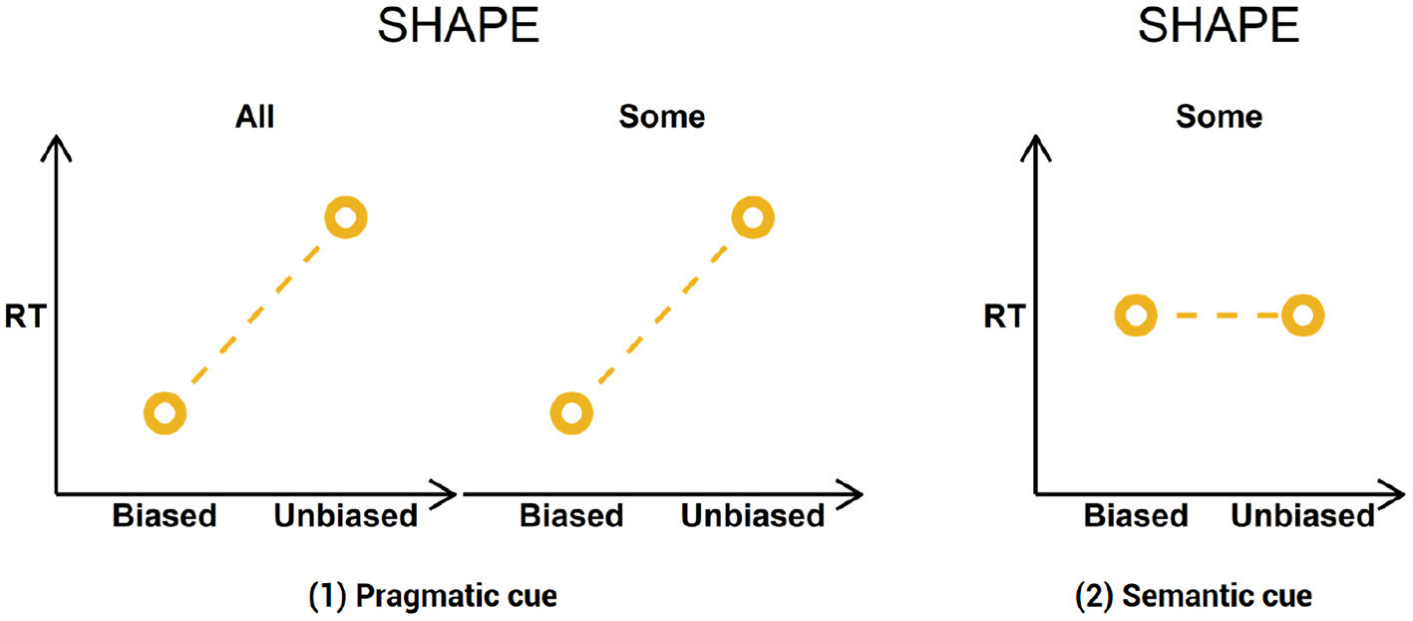
Study predictions.

A visual representation of the predictions is provided in Figure 5.

### 3.3. Results

#### 3.3.1. Data Cleaning

We recorded participants' reading times (RTs) as well as their sentence ratings. Two participants were excluded from the original sample due to faulty data recording which resulted in incomplete data sets, meaning that prior to data cleaning the sample consisted of 56 participants. For each test sentence, RTs were measured across all words, for a total of 8 to 10 measurement regions per sentence depending on the sentence type (descriptions referring to the position of the shapes contained two additional regions compared to the other two types of description). The data was cleaned according to two criteria: first, for any given trial, if the total RT differed by 2.5 positive or negative standard deviations from the mean total reading time for the respective condition, then the trial was excluded from any subsequent analysis; then, for any given participant, if their number of excluded trials was larger than 30% of the total number of trials, then the participant was excluded from any subsequent analysis. While no participant was excluded on the basis of these criteria, 776 individual trials were excluded from any subsequent analysis—amounting to a total of 0.02% of the relevant measurements. Moreover, inspection of the trial data inputted to the experimental program showed that in 1/3 of the Einige (False) items were coded erroneously, such that the critical term which rendered the descriptions false was the property term, when it should have been, in all cases, the shape term. In practice, what this particular sentence configuration did was to shift the incongruent critical term one region downstream. We therefore excluded the faulty trials from any subsequent analysis.

#### 3.3.2. Confirmatory Analyses

**Figure 6:**
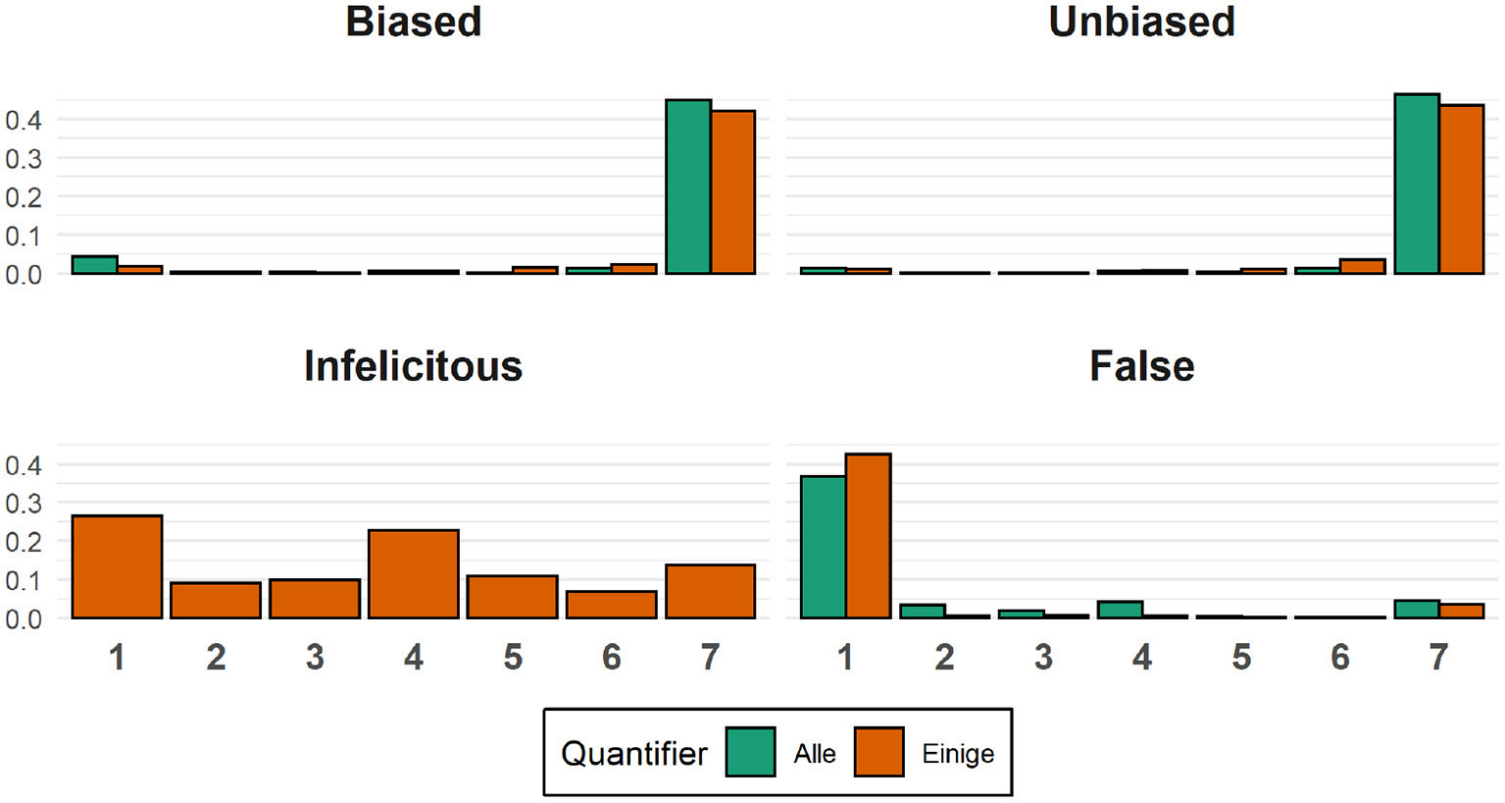
Sentence ratings. Each panel shows the proportion of ratings for a given critical condition. Ratings for sentences containing the quantifier *alle* are shown in green, while ratings for sentences containing the quantifier *einige* are shown in orange.

Recall that on each trial participants were asked to rate the accuracy of the description they read using a 7-point scale. In the case of the biased and unbiased conditions, all trials consisted of semantically congruent and thus, a priori, accurate image-description pairs. In the case of the false conditions, all trials consisted of semantically incongruent and thus, a priori, inaccurate image-description pairs. The distribution of participants' ratings can be seen in Figure 6 below:

Visual inspection of the graph suggests that only rarely did participants rate the semantically congruent expressions as inaccurate descriptions of the pictures (rating 1, 2, and 3 on the 7-point scale). Similarly, only rarely did they rate the semantically incongruent expressions as accurate descriptions of the pictures (rating 5, 6, and 7 on the 7-point scale). Note that the ratings for the Einige (Infelict) condition are somewhat evenly distributed over the whole scale, which means that there is wide variability in how participants interpreted descriptions containing the quantifier *einige*.

**Figure 7:**
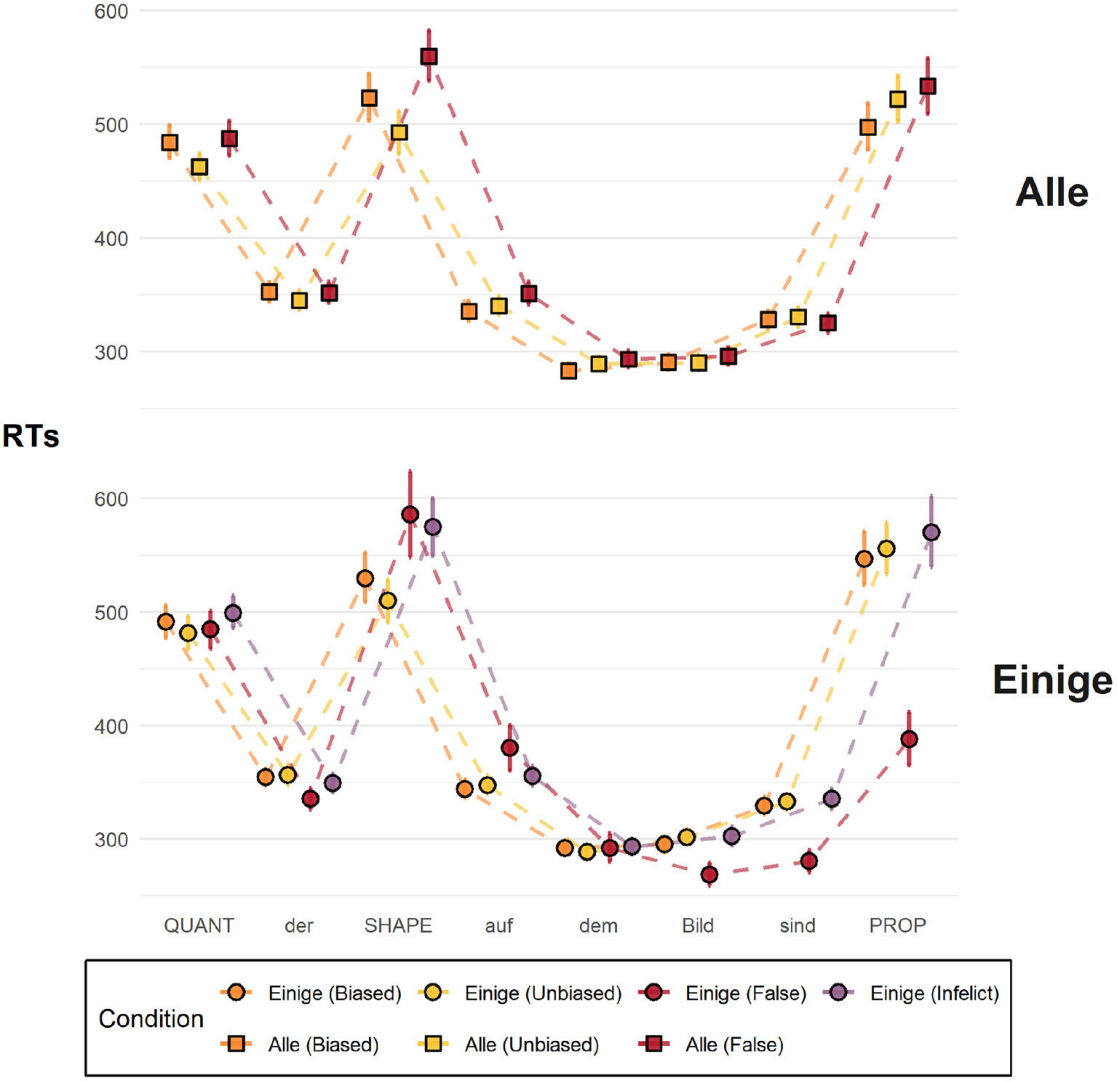
Mean reading times across all sentence regions up until the last critical term. The error bars represent 95% bootstrapped confidence intervals.

Below, in Figure 7, we plot the RT data. In order to amass quantitative evidence in favor of the reported results, we fitted Bayesian hierarchical models predicting RTs at the critical region, the SHAPE region, as a function of the experimental conditions, which themselves reflect different combinations of the quantifiers and the sentence bias. The models included, if possible, the maximal random effect structure justified by the design, which in our case is random intercepts for items—the actual pictures seen by participants, which varied systematically according to the experimental condition—and random slopes and intercepts for participants. Our models, fitted using the R package brms (Bürkner, 2017) and described in detail in the supporting material, had the following general form, shown in brms syntax:


$$\begin{array}{l} \log\left(\text{RT}\right)\sim \text{condition}+\\ \;\left(1+\text{condition}|\text{participant}\right)+\\ \;\left(1|\text{item}\right) \end{array}$$

For each highlighted result, we report whether or not the respective statistical model provides strong evidence in favor of the empirically attested differences (or lack thereof). In a Bayesian statistical framework, one is interested in the joint posterior distribution of the parameters of the model, which indicates a plausible range of values for the parameters given the model and the data at hand. We report a 95% credibility interval (CI) and the posterior probability that the parameter of interest, β, is larger than zero [*P*(β > 0)]. We speak of strong evidence for an effect when zero is not included in the CI and *P*(β > 0) is close to either zero or one. Concretely, we are interested in the difference between estimated values for cell means of conditions Einige (Biased) and Einige (Unbiased), as well as that between Alle (Biased) and Alle (Unbiased). Pragmatic surprisal theory predicts that, in the posterior distribution of the Bayesian regression model, the difference in cell means β__einige__ = [estimates for cell mean of Einige (Biased)] − [estimates for cell mean of Einige (Unbiased] should be credibly bigger than zero, so that *P*(β_einige_ > 0) should be large, i.e., very close to 1; similarly for the conditions with the quantifier *all*.

Figure 7 shows the mean RTs at each sentence region up until the last critical term, across all seven experimental conditions. QUANT, SHAPE, and PROPER are the critical regions where task-relevant information was read, namely, the quantifier, the shape term, and the property term, respectively. Visual inspection of the graph suggests that there are differences between the critical conditions and the control conditions, which serve as a diagnostic of participants' online sensibility to the semantic congruency of the descriptions. At the SHAPE region, participants read the critical term faster in the critical conditions [orange and yellow] compared to the their respective controls [red and purple] (for the regression coefficients, see the respective table in the supporting material), as is to be expected.

As for the critical condition-quantifier pairs, visual inspection of the graph suggests that there are differences at the SHAPE region. Interestingly, however, the results go in the opposite direction of the predictions of pragmatic surprisal theory, contradicting our hypothesis: we find strong evidence that in the case of both *einige* and *alle* participants took longer reading the shape term in the biased condition compared to the unbiased condition. Indeed, our statistical models show that there is practically no reason to believe that the RTs in the unbiased conditions are larger than those in the biased conditions (β_alle_ = −0.09, 95% CI [−0.14, −0.03], *P*(β_alle_ > 0) = 0.01; β_einige_ = −0.06, 95% CI [−0.11, 0.00], *P*(β_einige_ > 0) = 0.03).

#### 3.3.3. Summary

We found reliable differences in RTs at the SHAPE region, but these differences were in the opposite direction of what was predicted by pragmatic surprisal theory, which explains reading times as a monotone decreasing function of pragmatic expectability of the shape term in the context of the picture and the initial sentence segment. This theory predicts that both in the case of *einige* and *alle* RTs should be lower in the biased condition compared to the unbiased condition. However, in the case of both quantifiers, participants read the shape term more slowly in the biased condition compared to the unbiased condition. Table 2 summarizes the main results compared against the original predictions.

**Table 2:** Summary of the results.

	**Prediction**		**Result**	
	***Alle***	***Einige***	***Alle***	***Einige***
SHAPE	Biased < Unbiased	Biased < Unbiased	Biased > Unbiased	Biased > Unbiased

### 3.4. Discussion

All in all, the reported results warrant careful consideration. At face value, the observed pattern directly contradicts the predictions of pragmatic surprisal theory. But since PST consists of two components, the problem could lie with either component or both. Remember that PST assumes that (i) the reading times on a word are lower for more predictable words (the link function), and (ii) that probability of the next word is in turn influenced by contextual and pragmatic factors, in particular a preference for semantically true and pragmatically informative descriptions of the presented picture. Evidence against this conjunction of assumptions could be evidence against any one, or both, of these ideas.

Previous related work on pragmatic processing of scalar quantifiers in visually-anchored contexts (e.g., Spychalska et al., 2016; Augurzky et al., 2019) provides evidence for the idea that violations of pragmatic expectations, in the sense we are after here, do correspond with another assumed marker of next-word surprisal, namely the amplitude of an N400 component in ERPs (e.g., Kutas and Federmeier, 2011; Kuperberg and Jaeger, 2016). This suggests that context-induced pragmatic expectations, as conceived here, do seem to inform next-word expectations and may lead to surprisal-related processing difficulties.

There is also evidence that scalar implicature inferences (from *some* to *some but not all*) affect reading speed in self-paced reading studies. For example, Breheny et al. (2006) found increased reading times on a continuation with a phrase like *the rest* in contexts where a scalar implicature of a preceding occurrence of *some* was more expectable given a textual manipulation of expectation based on general world knowledge (similar results are presented by Bergen and Grodner, 2012). This suggests that self-paced reading times are, in principle, susceptible to pragmatic expectations of a sort.

#### 3.4.1. Processing Limitations

Given that there is some evidence for the ideas that (i) comprehenders do, at least sometimes, entertain pragmatic expectations of the kind of relevance here, induced by a visually presented context, and also that (ii) the self-paced reading method is susceptible to pragmatic factors, one possible explanation for our results could be that the specific combination of the kind of pragmatic expectations (induced by a context picture), on the one hand, and self-paced reading, on the other hand, does, for some reason or other, not work. It could, for example, be that since self-paced reading is a less natural way of reading than on-screen reading in rapid visual serial presentation (as used, e.g., in comparable EEG studies), the burden on working memory of remembering a complex picture, forming (pragmatic expectations) and reading text in a self-paced manner is too onerous a task. In fact, in a recent study employing mouse-tracking, Darley et al. (2020) found that processing sentences against a visual context becomes more costly the higher the number of pragmatically-licensed sentence continuations, such that, in their study, higher numbers of possible continuations led to a decrease in task accuracy, an increase in the speed of responses, as well as higher degrees of attraction to foil responses in the measured mouse trajectories. The authors concluded that “[…] the main effects of the number of possible sentence completions observed here constitute evidence that episodic associations may be less conducive to the rapid and incremental incorporation of information and associated prediction-making that is made possible by a rich pragmatic context (perhaps specifically relying on long-term semantic associations or world knowledge).” Though in a very different setup, our task also relies on episodic associations between visual stimuli and linguistic descriptions of those same stimuli, which is why it might be reasonable to raise similar issues in our case as well.

But even if limited processing resources are an issue, this does not straightforwardly reconcile our findings with pragmatic surprisal theory. The most natural effect of limited processing resources, on the assumption that the predictions of PST are basically correct, would arguably be that the predicted differences in RTs would be deflated, perhaps to the extent that they are completely unattested in the data, i.e., we should expect lesser or no differences where PST predicts differences. However, we see the exact opposite pattern in the data than what PST predicts. This is unexpected even if we make room for limitations of processing resources due to the complexity of the task. In conclusion, blaming limited processing resources does not seem to vindicate PST in the light of the observed data.

#### 3.4.2. Other Pragmatic Expectations

Pragmatic surprisal theory could be defended in the light of the obtained results by arguing that participants may have had different pragmatic expectations than the ones we assumed throughout. This, however, it not a very convincing position given that the very same participants showed production behavior in the first part of the experiment which supports very directly the kind of pragmatic expectations assumed in our formulation of PST's predictions.

#### 3.4.3. Task Effects

Another class of potential alternative explanations to consider is task effects. There are at least two different kinds of task effects. For one, participants might adapt gradually to the statistical properties of the experimental environment, e.g., learning to associate a particular type of display with a particular kind of sentence and likely response. For another, there are task effects which do not require knowledge of the statistics of the experimental environments but constitute an approximation to a rational solution to the task as presented in the instructions. These two types of task-induced effects differ with respect to when during the course of the experiment they arise. While the former, frequency-driven effects are expected to emerge later during the experiment as participants acquire knowledge of the relevant statistics, the latter effects can, in principle, be expected already early on in the experiment.

##### 3.4.3.1. Statistics of the Experimental Environment

**Figure 8:**
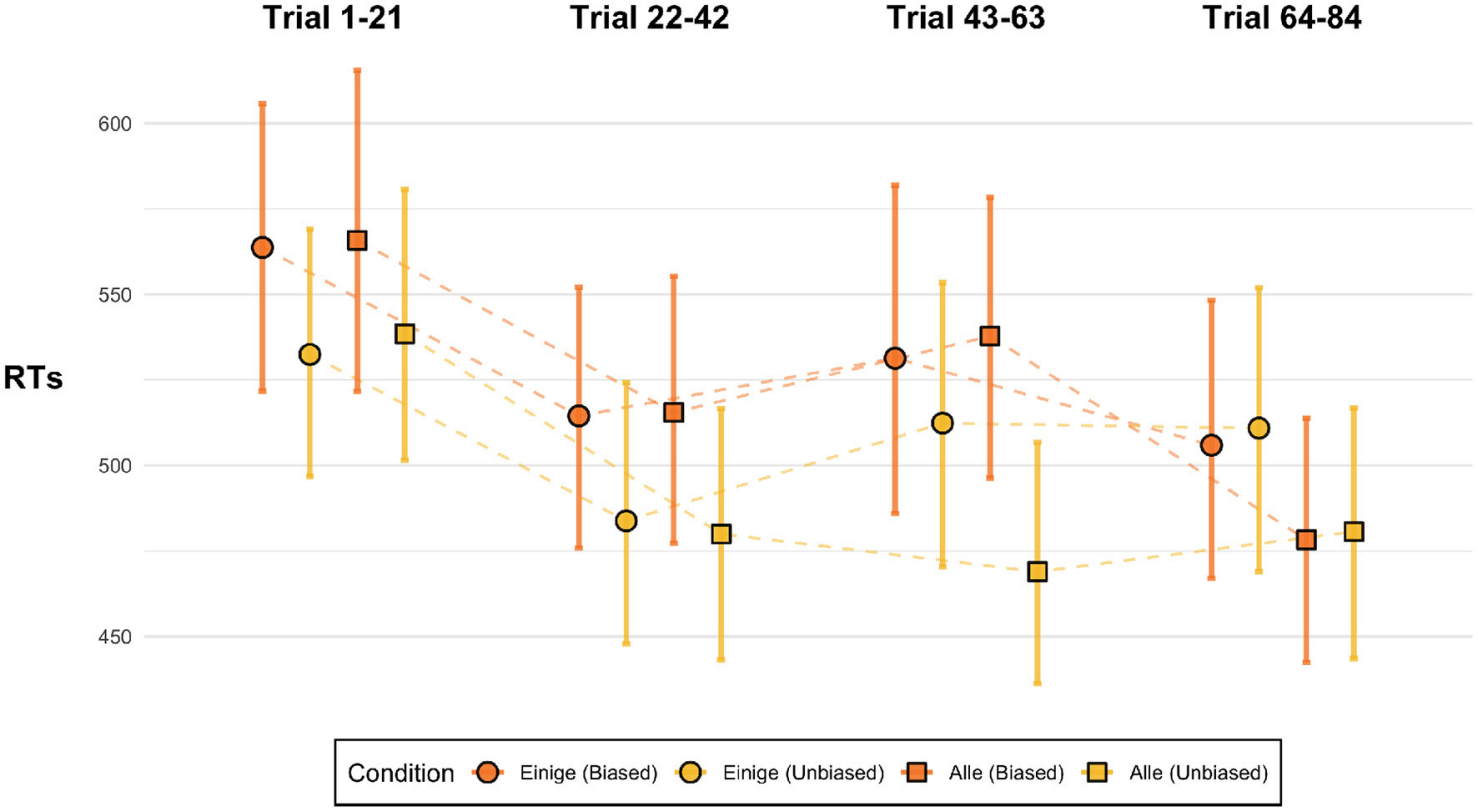
Reading times at the SHAPE region for the critical conditions for different blocks of the experiment. The dots show means, error bars are 95% bootstrapped confidence intervals.

Pragmatic surprisal theory would not be discredited by the pattern observed in the aggregate data if this overall pattern could plausibly be explained as a task-effect based on the statistics of the experimental environment. To vindicate PST in this way, the predictions of PST should be borne out in the early parts of the experiment even if later parts of the experiment show emerging adaptive strategies leading away from the predicted behavior of PST. However, as seen in Figure 8, already during the first block of the experiment the main effect we see for the aggregate data—the opposite of what PST would predict—shows in the mean reading times (the Supplementary Material provides in-depth *post-hoc* analysis of block effects). Indeed, at least numerically, the biased conditions are faster than the unbiased conditions across the whole experiment except the last block. There is no support for PST at the beginning of the experiment, nor in any other block. This suggests that vindicating PST by appeal to task effects that hinge on participants adapting to the statistics of the experimental environment is not very plausible.

##### 3.4.3.2. Strategic Allocation of Attentional Resources

It remains to speculate about alternative *post-hoc* explanations based on optimal solutions to the task without knowledge of the statistics of the environment. One conceivable alternative explanation for the faster reading of the unbiased conditions, compared to the biased conditions, revolves around *strategic allocation of attentional resources*. In this picture, what matters to self-paced reading speed is the immediate relevance of a chunk or word to the assessment of the pragmatic felicity of the unfolding descriptions. In other words, participants may be said to have read the shape terms more carefully, and thus more slowly, if the information at a given sentence region was relevant for assessing the pragmatic felicity of the description.

Consider, for instance, a scenario composed of a homogeneous triangle array and a heterogeneous circle array (Figure 4A; cf. Figure 4B). Having read *Einige der* (English: Some of the), even though a participant might be biased, as per the design, to expect *circles* next, she needs to know exactly whether *triangles* or *circles* are actually referred to—if *triangles*, the description is very likely to be either underspecified or downright false; if *circles*, then the load of determining pragmatic felicity is shifted to the subsequent critical region. Similarly, in the same scenario composed of a homogeneous triangle array and a heterogeneous circle array, having read *Alle der* (English: All of the), even though a participant might be biased, by design, to expect *triangles*, the shape term is key in determining whether the unfolding description is congruent or not: if *circles* are referred to, the description is rendered false at that very sentence region; if, instead, *triangles* are referred to, then the load of determining the congruency of the description is shifted to the subsequent critical region.

Strategic allocation of attentional resources might explain the observed difference between unbiased conditions (read fast because they are irrelevant to the truth-judgement of the sentence) and biased conditions (potentially relevant information at the shape position to the truth-judgement of the sentence). This idea also explains why Einige (False) is read faster than Einige (Infelicitous). Notice that the context picture associated with Einige (False) is the same as that for Einige (Unbiased). Of course, to explain the increased reading times for conditions where the shape term makes the sentence (most likely) false, possibly by implicature, this alternative explanation must also stipulate a reading time increment for falsity.

## 4. Summary and Conclusion

This paper presented the results of a study designed to test the predictions of pragmatic surprisal theory. According to PST, visually-anchored contexts should induce pragmatic expectations about next-word continuations of sentences, and these pragmatic expectations should lead to increased processing efforts proportional to how unexpected incoming linguistic material is. PST is supported by previous research using EEG (e.g., Spychalska et al., 2016; Augurzky et al., 2019). As an alternative line of research also links next-word suprisal to reading times (e.g., Demberg and Keller, 2008; Fernandez Monsalve et al., 2012; Smith and Levy, 2013), the study presented here aimed to test PST in the context of a self-paced reading study with visually-anchored contexts.

The observed results are in clear conflict with the predictions of PST. While limitations of processing resources might be relevant for our particular experimental design, it is not obvious how taking these into account would reconcile our empirical findings with PST. Based on *post-hoc* inspection of the temporal development over the course of the experiment, we argue that it is unlikely that the observed pattern, which we interpret as evidence against PST, is a task effect driven by the statistics of the experimental environment. We suggest an alternative *post-hoc* explanation according to which reading times are a function of the strategic allocation of attentional resources, a process which is itself informed by context-induced pragmatic considerations but which does not rely on knowledge of the statistics of the experimental environment.

Ultimately, then, our results seem to suggest that predictability in online pragmatic processing is linked not only to purely pragmatic-based prediction but also to other processing constraints, such as, in our case, one derived from a pressure to integrate crucial semantic information incrementally during processing. This is in line with constraint-based accounts of language comprehension which assume not only that multiple sources of information need to be integrated online during processing but perhaps more importantly that the weight of different constraints varies depending on the specific processing demands as well as on the larger discourse and communicative context (Degen and Tanenhaus, 2015). In fact, other studies on online pragmatic processing have also shown that pragmatic inferencing, including scalar inferencing cued by quantifiers, shows variable time courses and strong context-dependence (Bergen and Grodner, 2012; Urbach et al., 2015; Huang and Snedeker, 2018). What these different studies seem to agree on is that there is immense variability in how pragmatic interpretation works, from its contexts of occurrence, to its online signatures, and ultimately its underlying mechanistic processes. We conclude that more research is necessary to investigate how pragmatic expectations—as captured by the construal of PST formulated and tested here—combine or interact with resourceful task-dependent processing strategies.

## Data Availability Statement

The datasets presented in this study can be found in online repositories. All materials (data, materials, scripts, figures, and supplementary figures) associated with this manuscript are available on https://doi.org/10.5281/zenodo.5156186.

## Ethics Statement

The studies involving human participants were reviewed and approved by Ethics committee of the Fachbereich 8 of the Osnabrück University. The patients/participants provided their written informed consent to participate in this study.

## Author Contributions

VM and MF planned the research and wrote the paper. VM realized and conducted the experiment and analyzed the data. MF assisted in analyzing the data. Both authors contributed to the article and approved the submitted version.

## Conflict of Interest

The authors declare that the research was conducted in the absence of any commercial or financial relationships that could be construed as a potential conflict of interest.

## Publisher's Note

All claims expressed in this article are solely those of the authors and do not necessarily represent those of their affiliated organizations, or those of the publisher, the editors and the reviewers. Any product that may be evaluated in this article, or claim that may be made by its manufacturer, is not guaranteed or endorsed by the publisher.

## Footnotes

^1^The semantic incongruency of these sentences arguably hinges on a presupposition failure rather than false *at-issue* content. From the point of view of expectation-based language processing, this formal difference should, however, make little difference since the term “Quadrate” will be very unexpected for the picture and participants are very unlikely to choose this term as a viable description of the picture.

^2^The experimental program is available for inspection under https://spr-in-lab-june-2019-direct-folder.netlify.com/.
