# Corrigendum: Pragmatic Prediction in the Processing of Referring Expressions Containing Scalar Quantifiers

**DOI:** 10.3389/fpsyg.2021.775007

**Published:** 2021-09-30

**Authors:** Vinicius Macuch Silva, Michael Franke

**Affiliations:** Cognitive Modeling Group, Institute of Cognitive Science, Osnabrück University, Osnabrück, Germany

**Keywords:** pragmatics, predictive procesing, Pragmatic prediction, Scalar quantifiers, self-paced reading, German

In the original article, there was an error in the Data Availability Statement as published. The link to the repository with the open materials was missing from the original statement.

A correction has been made to *Data Availability Statement*. The corrected statement is shown below.

The datasets presented in this study can be found in online repositories. All materials (data, materials, scripts, figures, and supplementary figures) associated with this manuscript are available on https://doi.org/10.5281/zenodo.5156186.

The authors apologize for this error and state that this does not change the scientific conclusions of the article in any way. The original article has been updated.

## Publisher's Note

All claims expressed in this article are solely those of the authors and do not necessarily represent those of their affiliated organizations, or those of the publisher, the editors and the reviewers. Any product that may be evaluated in this article, or claim that may be made by its manufacturer, is not guaranteed or endorsed by the publisher.

