# Supplementary material for: Pragmatic Prediction in the Processing of Referring Expressions Containing Scalar Quantifiers
Source: Front Psychol. 2021 Aug 31;12:662050. doi: 10.3389/fpsyg.2021.662050 (PMC8438145; doi:10.3389/fpsyg.2021.662050)
Supplement: Supplementary file 1 [file Data_Sheet_1.PDF]

Supplementary Material

In the following we supplement the main analyses with additional *post hoc* analyses, by additionally considering potential effects of the shape terms, the experimental block, and the "pragmatic profile" of participants, i.e., whether a participant gave answers consistent with a pragmatic interpretation of *some*.

1 EFFECTS OF SHAPE TERM

In order to explore whether there are further processing differences depending on the particular critical word participants read each trial, we analyze the RT data according to the different shape terms found in the experiment, namely *Dreiecke* (triangles), *Kreise* (circles), and *Quadrate* (squares). We fitted a hierarchical model predicting RTs at the SHAPE region as a function of both the experimental conditions and the shape terms, including random intercepts for items and random slopes and intercepts for participants, as shown in the `brms` syntax below:

$$\begin{aligned}\text{log(RT)} \sim \text{condition * shape term} +\\ &(1 + \text{condition} + \text{shape term} \mid \text{participant}) +\\ &(1 \mid \text{item})\end{aligned}$$

Figure S3 shows the mean RTs at the SHAPE region for each shape term. Visual inspection of the graph suggests that the comparisons of interest – between the biased and unbiased conditions for each quantifier – pattern similarly to the aggregate data, the results being, overall, not in line with the predictions of pragmatic surprisal theory. In the case of all three shape terms we find no evidence that participants took longer reading the critical word in the Einige (Unbiased) condition compared to the Einige (Biased) condition (*Dreiecke*: β_{einige} = -0.02, 95% CI [-0.09, 0.04], $P(\beta_{\text{einige}} > 0) = 0.29$; *Kreise*: β_{einige} = -0.05, 95% CI [-0.13, 0.03], $P(\beta_{\text{einige}} > 0) = 0.16$; *Quadrate*: β_{einige} = -0.03, 95% CI [-0.11, 0.05], $P(\beta_{\text{einige}} > 0) = 0.27$). As for *alle*, we find no evidence that participants took longer reading either *Kreise* or *Quadrate* in the unbiased condition compared to the biased condition (*Kreise*: β_{alle} = -0.06, 95% CI [-0.12, 0.00], $P(\beta_{\text{alle}} > 0) = 0.06$; *Quadrate*: β_{alle} = -0.04, 95% CI [-0.10, 0.02], $P(\beta_{\text{alle}} > 0) = 0.12$), while there is strong evidence that they took longer reading *Dreiecke* in the biased condition (*Dreiecke*: β_{alle} = -0.12, 95% CI [-0.20, -0.03], $P(\beta_{\text{alle}} > 0) = 0.01$). Figure S2 below shows a visual summary of all model estimates and their respective credible intervals.

Hypothesis	Estimate	Est.Error	CI.Lower	CI.Upper	Evid.Ratio	Post.Prob
**Einige**						
[Dreiecke] Unbiased >Biased	-0.02	0.04	-0.09	0.04	0.41	0.29
[Kreise] Unbiased >Biased	-0.05	0.05	-0.13	0.03	0.19	0.16
[Quadrate] Unbiased >Biased	-0.03	0.05	-0.11	0.05	0.36	0.27
**Alle**						
[Dreiecke] Unbiased >Biased	-0.12	0.05	-0.20	-0.03	0.01	0.01
[Kreise] Unbiased >Biased	-0.06	0.04	-0.12	0.00	0.07	0.06
[Quadrate] Unbiased >Biased	-0.04	0.04	-0.10	0.02	0.14	0.12

Table S1. Model coefficients.

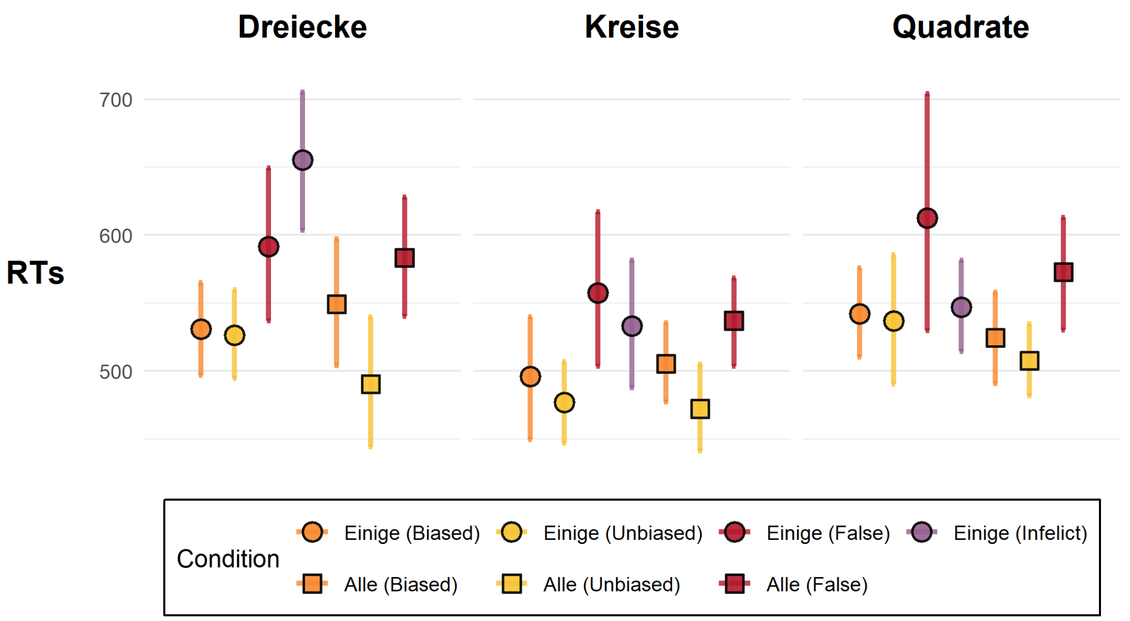

Figure S1. Mean reading times at the SHAPE region by shape term. The error bars represent 95% bootstrapped confidence intervals.

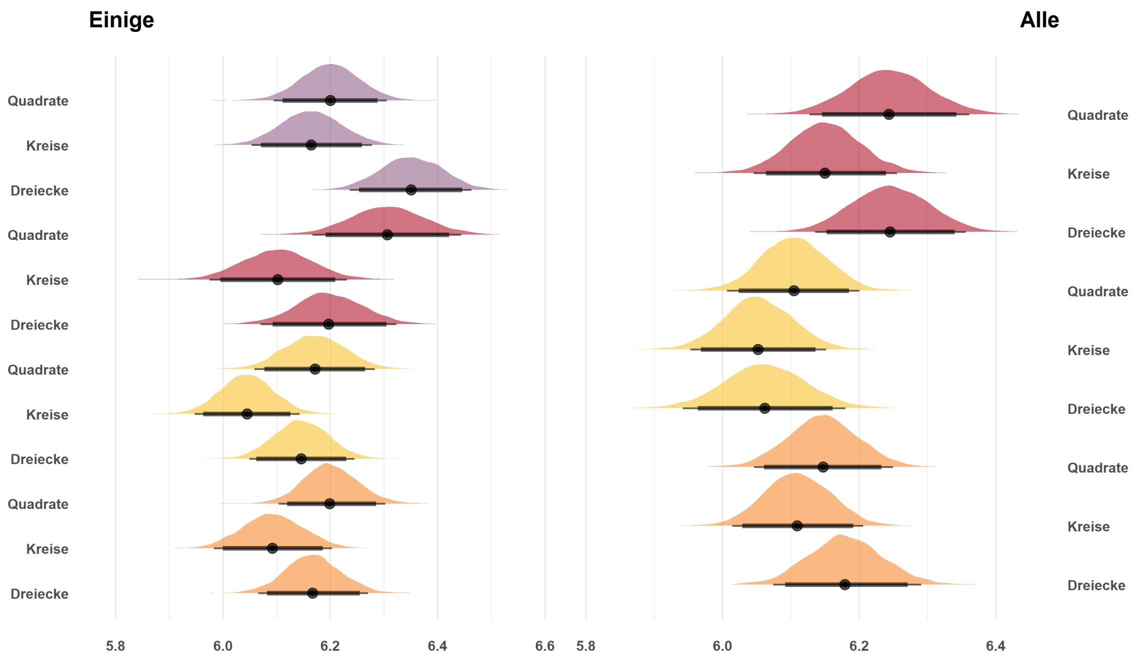

Figure S2. Posterior estimates and credible intervals. The thick and thin black horizontal lines show 90% and 95% uncertainty over the model estimates, respectively.

2 EFFECTS OF EXPERIMENTAL BLOCK

In order to explore whether there are possible statistical learning effects during the experiment, such that the processing patterns found in the aggregate data only emerge after participants learn the distributional properties of the experimental items, we analyze the RT data in terms of four experimental blocks, each block encompassing 21 trials. We fitted a hierarchical model predicting RTs at the SHAPE region as a function of both the experimental conditions and the experimental blocks, including random slopes and intercepts for both items and participants, as shown in the `brms` syntax below:

$$\begin{aligned}\text{log(RT)} \sim\ & \text{condition * block +}\\ & \text{(1 + condition + block | participant) +}\\ & \text{(1 + block | item)}\end{aligned}$$

Figure S3 shows the mean RTs at the SHAPE region for each experimental block. Visual inspection of the graph suggests that the comparisons of interest – between the biased and unbiased conditions for each quantifier – pattern similarly to the aggregate data, the results being, overall, not in line with the predictions of pragmatic surprisal theory. In the first two experimental blocks we find strong evidence *against* the original predictions, such that participants took longer reading the critical word in the Einige (Biased) condition compared to the Einige (Unbiased) condition (Trial 1-21: β_{einige} = -0.08, 95% CI [-0.15, -0.01], $P(\beta_{\text{einige}} > 0) = 0.04$; Trial 22-42: β_{einige} = -0.08, 95% CI [-0.15, 0.00], $P(\beta_{\text{einige}} > 0) = 0.04$). In the last two blocks, we find no evidence that participants took longer reading the critical word in Einige (Unbiased) compared to Einige (Unbiased) (Trial 43-3: β_{einige} = -0.03, 95% CI [-0.10, 0.05], $P(\beta_{\text{einige}} > 0) = 0.30$; Trial 64-84: β_{einige} = 0.01, 95% CI [-0.056, 0.09], $P(\beta_{\text{einige}} > 0) = 0.61$). As for *alle*, across all blocks except the third one we find no evidence that participants took longer reading the critical word in the unbiased condition compared to the biased condition (Trial 1-21: β_{alle} = -0.07, 95% CI [-0.14, 0.00], $P(\beta_{\text{alle}} > 0) = 0.53$; Trial 22-42: β_{alle} = 0.07, 95% CI [-0.15, 0.00], $P(\beta_{\text{alle}} > 0) = 0.55$; Trial 64-84: β_{alle} = -0.03, 95% CI [-0.10, 0.04], $P(\beta_{\text{alle}} > 0) = 0.25$). In the case of the third block we find strong evidence that participants took longer reading the critical word in the biased condition (Trial 43-63: β_{alle} = -0.08, 95% CI [-0.15, -0.01], $P(\beta_{\text{alle}} > 0) = 0.03$). Figure S4 below shows a visual summary of all model estimates and their respective credible intervals.

Hypothesis	Estimate	Est.Error	CI.Lower	CI.Upper	Evid.Ratio	Post.Prob
**Einige**						
[1-21] Unbiased >Biased	-0.08	0.04	-0.15	-0.01	0.04	0.04
[22-42] Unbiased >Biased	-0.08	0.05	-0.16	0.00	0.05	0.04
[43-63] Unbiased >Biased	-0.03	0.05	-0.10	0.05	0.43	0.30
[64-84] Unbiased >Biased	0.01	0.05	-0.06	0.09	1.59	0.61
**Alle**						
[1-21] Unbiased >Biased	-0.07	0.04	-0.14	0.00	0.06	0.05
[22-42] Unbiased >Biased	-0.07	0.05	-0.15	0.00	0.06	0.06
[43-63] Unbiased >Biased	-0.08	0.04	-0.15	-0.01	0.03	0.03
[64-84] Unbiased >Biased	-0.03	0.04	-0.10	0.04	0.33	0.25

Table S2. Model coefficients.

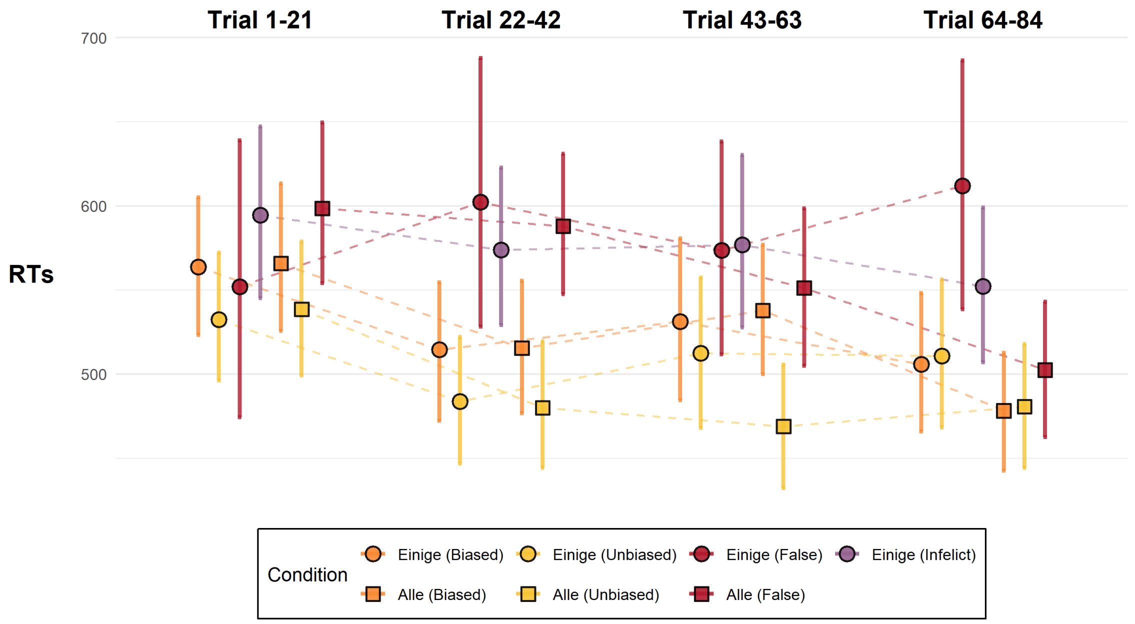

Figure S3. Mean reading times at the SHAPE region by experimental block. The error bars represent 95% bootstrapped confidence intervals.

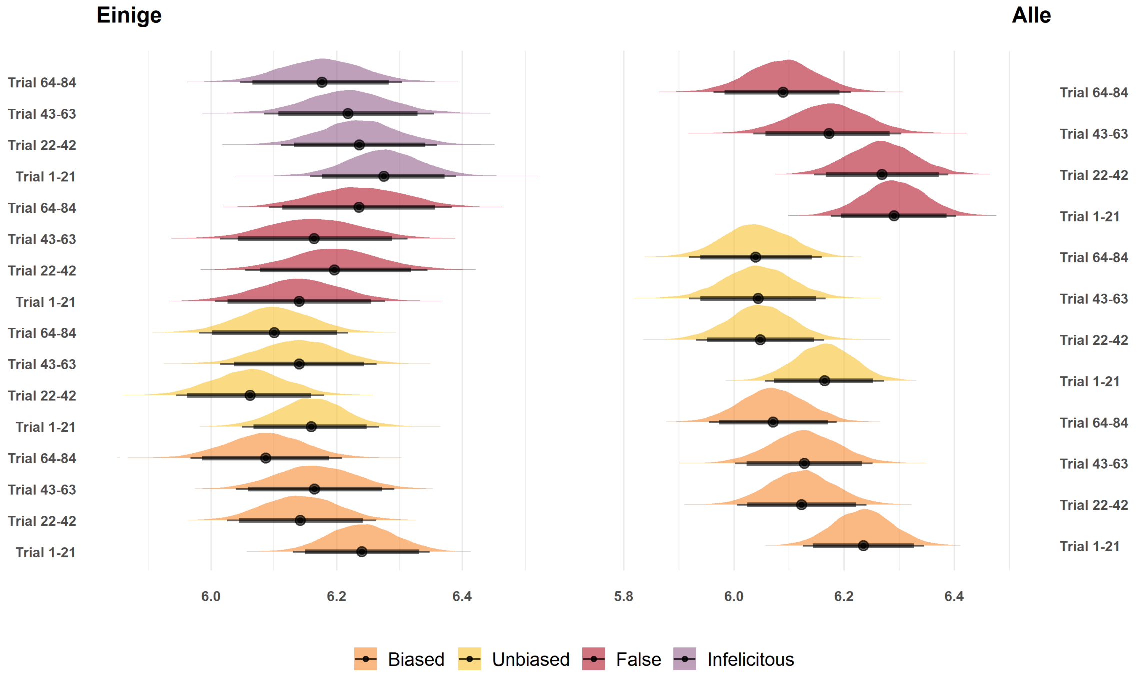

Figure S4. Posterior estimates and credible intervals. The thick and thin black horizontal lines show 90% and 95% uncertainty over the model estimates, respectively.

3 EFFECTS OF PRAGMATIC PROFILE

In order to explore whether there are further processing differences depending on the pragmatic profile exhibited by participants, we analyze the RT data according to how participants responded to the comprehension questions on the pragmatically infelicitous trials. We divide participants into those who always rated infelicitous expressions as accurate scene descriptions (semantic respondents, n = 6/ 56), those who always rated those same expressions as inaccurate scene descriptions (pragmatic respondents, n = 10/ 56), and those who show a mixed response pattern (mixed respondents, n = 40/ 56). We fitted three hierarchical models predicting RTs at the SHAPE region, one base model having both experimental condition and pragmatic profile as predictors, one including shape term as an additional predictor, and one including experimental block instead of shape term as an additional predictor. Since we classify each participant as belonging to exactly one pragmatic profile and since each item belongs, by design, to exactly one experimental condition, our models include random intercepts for both items and participants. The base model is shown in the `brms` syntax below:

$$\begin{aligned} \text{log(RT)} \sim \text{condition * pragmatic profile +} \\ \text{(1 + condition} \mid \text{participant) +} \\ \text{(1 + pragmatic profile} \mid \text{item)} \end{aligned}$$

Figure S5 shows the mean RTs at the SHAPE region according to each respondent type. Visual inspection of the graph suggests that there is variation between the critical condition-quantifier pairs across the different profiles. We find no evidence that semantic respondents read the critical word more slowly in the Alle (Unbiased) condition [yellow square] compared to Alle (Biased) [orange square] (β_{alle} = -0.11, 95% CI [-0.23, 0.01], $P(\beta_{\text{alle}} > 0) = 0.07$), while there is strong evidence that pragmatic respondents read the critical word more slowly in the unbiased condition (β_{alle} = -0.15, 95% CI [-0.25, -0.06], $P(\beta_{\text{alle}} > 0) = 0.01$). As for the *einige* conditions [circles], we find no evidence that either pragmatic or semantic respondents read the shape term more slowly in the unbiased condition [yellow circle] (semantic: β_{einige} = -0.06, 95% CI [-0.21, 0.08], $P(\beta_{\text{einige}} > 0) = 0.25$; pragmatic: β_{einige} = -0.06, 95% CI [-0.17, 0.05], $P(\beta_{\text{einige}} > 0) = 0.19$). As for mixed respondents, again we find no evidence of an effect in the case of both critical condition-quantifier pairs (see below for regression coefficients). These results conflict with the prediction that $\text{RT}_{\text{Einige (Biased)}}$ is smaller than $\text{RT}_{\text{Einige (Unbiased)}}$, when considering pragmatic respondents, though they are in line with the prediction that $\text{RT}_{\text{Einige (Biased)}}$ equals $\text{RT}_{\text{Einige (Unbiased)}}$, when considering semantic respondents. Figure S6 below shows a visual summary of all model estimates and their respective credible intervals.

Hypothesis	Estimate	Est.Error	CI.Lower	CI.Upper	Evid.Ratio	Post.Prob
**Einige**						
[semantic] Unbiased >Biased	-0.06	0.09	-0.21	0.08	0.33	0.25
[pragmatic] Unbiased >Biased	-0.06	0.07	-0.17	0.05	0.23	0.19
[mixed] Unbiased >Biased	-0.05	0.04	-0.11	0.01	0.08	0.07
**Alle**						
[semantic] Unbiased >Biased	-0.11	0.07	-0.23	0.01	0.07	0.07
[pragmatic] Unbiased >Biased	-0.15	0.06	-0.25	-0.06	0.01	0.01
[mixed] Unbiased >Biased	-0.03	0.03	-0.08	0.02	0.22	0.18

Table S3. Model coefficients.

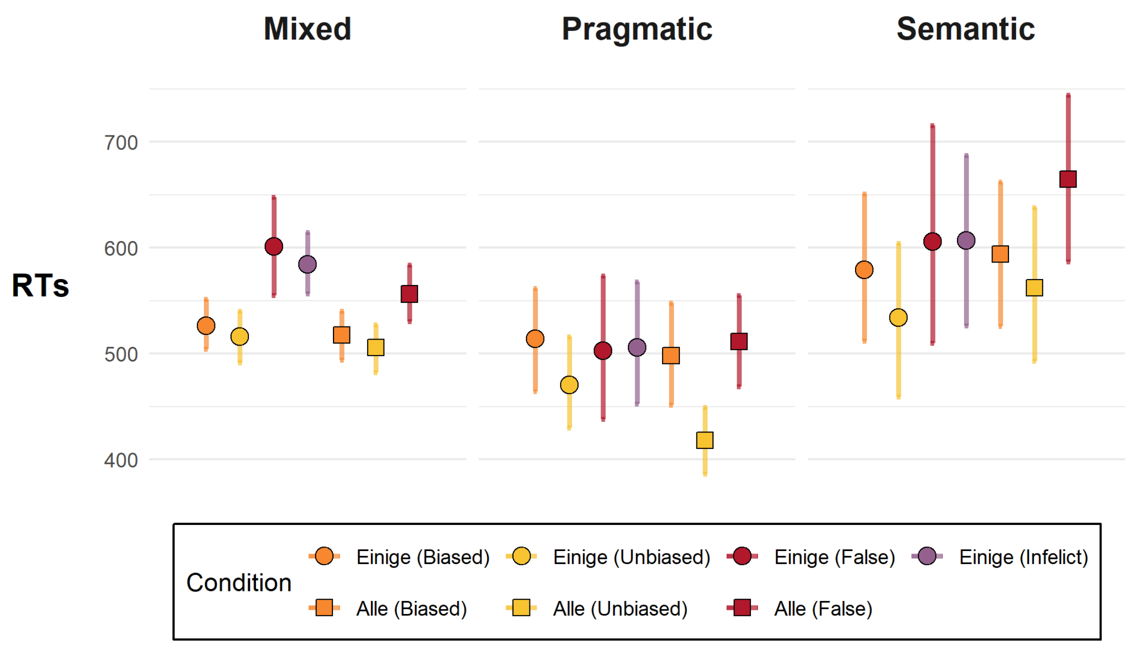

Figure S5. Mean reading times at the SHAPE region by pragmatic profile. The error bars represent 95% bootstrapped confidence intervals.

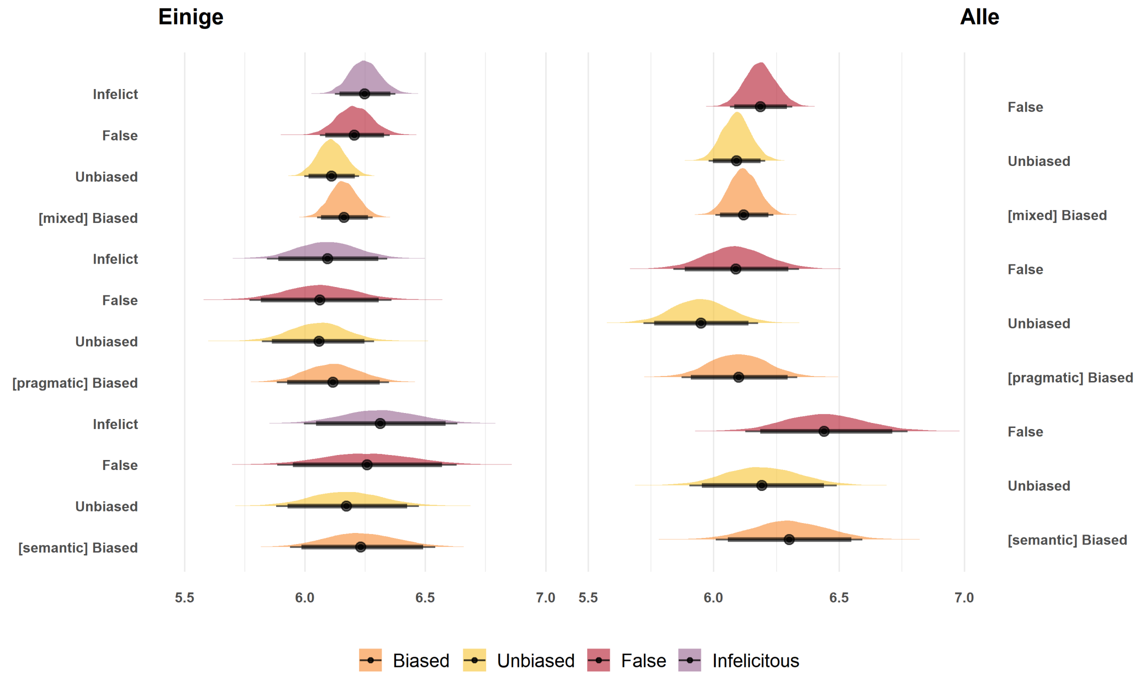

Figure S6. Posterior estimates and credible intervals for the model predicting RTs as a function of the experimental conditions and pragmatic profile. The thick and thin black horizontal lines show 95% and 90% uncertainty over the model estimates, respectively.

We also report two additional analyses, one derived from a model including critical word as an additional predictor, and another derived from a model including experimental block as an additional predictor, as shown in the `brms` syntax below:

$$
\begin{aligned}
\log(\text{RT}) \sim\ & \text{condition * pragmatic profile * shape term +} \\
& \text{(1 + condition + critical} \mid \text{participant) +} \\
& \text{(1 + pragmatic profile} \mid \text{item)}
\end{aligned}
$$

$$
\begin{aligned}
\log(\text{RT}) \sim\ & \text{condition * pragmatic profile * block +} \\
& \text{(1 + condition + block} \mid \text{participant) +} \\
& \text{(1 + pragmatic profile} \mid \text{item)}
\end{aligned}
$$

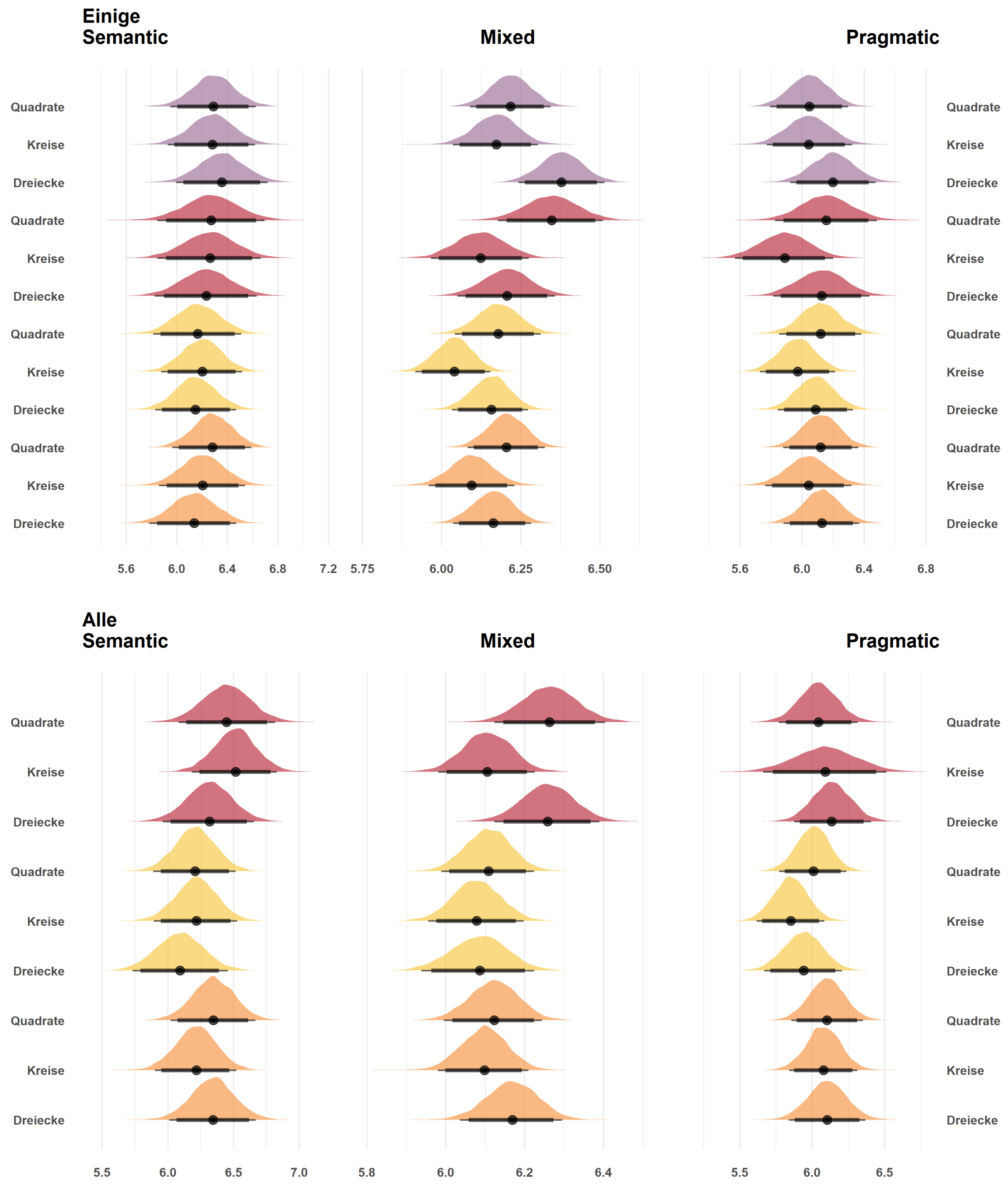

Figure S7. Posterior estimates and credible intervals for the model predicting RTs as a function of the experimental conditions, pragmatic profile, and critical word. The thick and think black horizontal lines show 90% and 95% uncertainty over the model estimates, respectively.

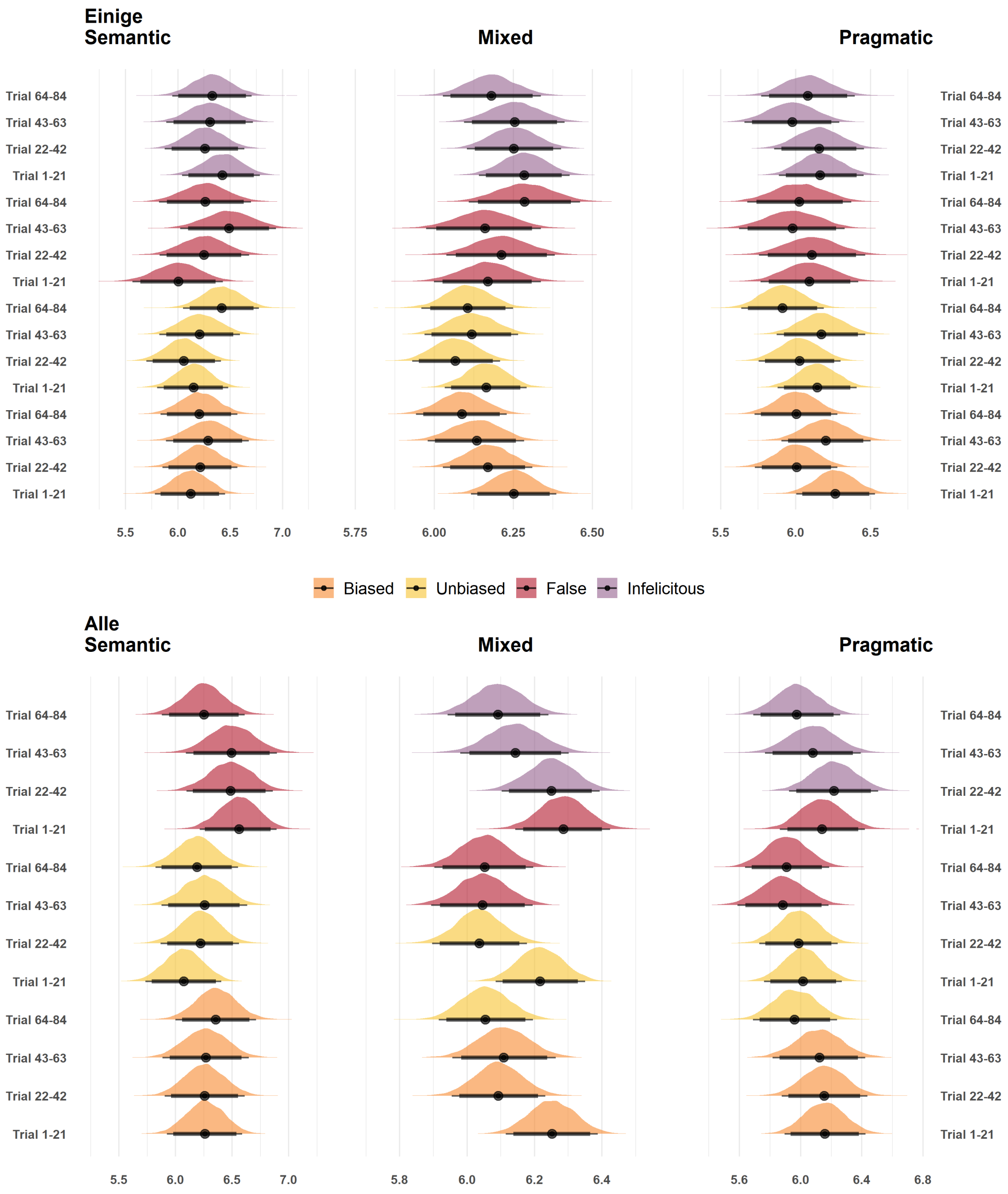

Figure S8. Posterior estimates and credible intervals for the model predicting RTs as a function of the experimental conditions, pragmatic profile, and experimental block. The thick and thin black horizontal lines show 90% and 95% uncertainty over the model estimates, respectively.
